# SOCIAL ISOLATION, LONELINESS, AND DEPRESSION IN FALLS AMONG RACIALLY/ETHNICALLY DIVERSE OLDER AMERICANS

**DOI:** 10.1093/geroni/igae098.2721

**Published:** 2024-12-31

**Authors:** Ayse Malatyali, Lisa Wiese, Tom Cidav, Rui Xie, Ladda Thiamwong

**Affiliations:** University of Central Florida, Orlando, Florida, United States; Florida Atlantic University, Boca Raton, Florida, United States; U.S. Department of Veterans Affairs (VA), Philadelphia, Pennsylvania, United States; University of Central Florida, Orlando, Florida, United States; University of Central Florida, Orlando, Florida, United States

## Abstract

Social isolation and loneliness affect 20%-30% of older Americans, and they increase the risk of depression, admission to nursing homes, early disability, and mortality. Poor social networks and social relations are also related to falls, but research on social isolation and loneliness in falls is limited. This study examines the association of social isolation, loneliness, and depression with falls among older Americans. Using logistic regression analyses, we analyzed data from the Health Retirement Study (2020 interviews) cross-sectionally. Measures included the HRS fall questionnaire, five-item social isolation scale, UCLA loneliness scale, and Center for Epidemiological Studies Depression scale. Our sample included 8,946 participants (age>64) with a mean age of 76. 59% of the participants were women, 26.3% living in rural, and 36% reported a fall experience in the past. The likelihood of having a fall was 10% higher in participants with higher levels of social isolation and 40% higher in participants with loneliness than those who were not socially isolated or lonely. Participants with depressive symptoms were 85% more likely to have a fall compared to participants without depressive symptoms. African Americans were 23% less likely to have a fall than Whites. The likelihood of having a fall was 19% less in men than women. Participants living in rural areas were 15% more likely to have a fall than participants living in urban areas. Further investigation into the interaction between social isolation, loneliness, and demographics is needed. Falls prevention interventions should include African Americans, women, and older adults in rural areas.

